# Editorial: Advances and Challenges of RNAi Based Technologies for Plants

**DOI:** 10.3389/fpls.2021.680242

**Published:** 2021-05-07

**Authors:** Bruno Mezzetti, Matthias Fladung, Jeremy Sweet

**Affiliations:** ^1^Department of Agriculture, Food and Environmental Sciences, Università Politecnica delle Marche, Ancona, Italy; ^2^Thünen-Institute of Forest Genetics, Grosshansdorf, Germany; ^3^Environmental Consultant, Cambridge, United Kingdom

**Keywords:** gene silencing (siRNA), cross kingdom, pathogen control, biosafety, sustainable agriculture

In this Research Topic, the focus is on recent research and developments in the use of RNAi techniques to protect plants. Cross kingdom effects of RNA expressed in plants primarily for pathogen control are discussed by Schaefer et al. in *Cross-Kingdom RNAi of Pathogen Effectors Leads to Quantitative Adult Plant Resistance in Wheat*.

Pest as well as pathogen control is considered in *Plant miRNA Cross-Kingdom Transfer Targeting Parasitic and Mutualistic Organisms as a Tool to Advance Modern Agriculture* by Gualtieri et al..

Bachman et al. consider aspects of their research on dsRNA expressed in maize/corn for root worm beetle control in *Sequence–Activity Relationships for the Snf7 Insecticidal dsRNA in Chrysomelidae*.

The efficiency and efficacy of a system for lepidopteran control is discussed in *Comparative Analysis of Chitin SynthaseA dsRNA Mediated RNA Interference for Management of Crop Pests of Different Families of Lepidoptera* by Rana et al..

The potential for plants to express RNA viruses which can infect both plants and insects but which are pathogenic in their insect vectors is considered as a strategy for vector insect control in *The Use of Engineered Plant Viruses in a Trans-Kingdom Silencing Strategy Against Their Insect Vectors* by Kolliopoulou et al..

The biosafety assessments of these and other host induced gene silencing (HIGS) systems are discuss in *Biosafety of GM Crop Plants Expressing dsRNA: Data Requirements and EU Regulatory Considerations* by Arpaia et al..

Exogenous application of dsRNA (SIGS) for pest and pathogen control is an attractive alternative but is confronted with several problems related to application, uptake, persistence, and efficacy. In *Barriers to Efficient Foliar Uptake of dsRNA and Molecular Barriers to dsRNA Activity in Plant Cells*, these are discussed by Bennett et al..

Uslu et al. describe studies of high pressure spraying to improve efficacy in *High-Pressure-Sprayed Double Stranded RNA Does Not Induce RNA Interference of a Reporter Gene*.

In relation to virus control, Tabein et al. discuss *The Induction of an Effective dsRNA-Mediated Resistance Against Tomato Spotted Wilt Virus by Exogenous Application of Double-Stranded RNA Largely Depends on the Selection of the Viral RNA Target Region*.

Research on control of a major insect pest is reviewed in *Validating the Potential of Double-Stranded RNA Targeting Colorado Potato Beetle Mesh Gene in Laboratory and Field Trials* by Petek et al..

These papers give an insight into the many areas of research on the potential applications for RNAi for crop protection. Some applications are being commercialized while others are at earlier stages of research (Taning et al., 2020a). The Editors believe that applications of HIGS and SIGS can make major contributions to sustainable and integrated crop protection and support “Green” systems for intensification of agricultural production as advocated in UN Sustainability Development Goals and by FAO, EU, and many national governments (Mezzetti et al., 2020; Taning et al., 2020b). The editors would like to acknowledge the opportunity provided by Frontiers for publishing these papers and thank the authors for their contributions. The editors and many of the authors are members of the iPlanta COST action CA 15223 and further information on iPlanta and publications produced by iPlanta are available at https://iplanta.univpm.it/.

## Author Contributions

All authors listed have made a substantial, direct and intellectual contribution to the work, and approved it for publication.

## Conflict of Interest

The authors declare that the research was conducted in the absence of any commercial or financial relationships that could be construed as a potential conflict of interest.
